# Parent and Teacher-Reported Child Outcomes Seven Years After Mild Traumatic Brain Injury: A Nested Case Control Study

**DOI:** 10.3389/fneur.2021.683661

**Published:** 2021-07-23

**Authors:** Kelly M. Jones, Nicola Starkey, Suzanne Barker-Collo, Shanthi Ameratunga, Alice Theadom, Katy Pocock, Robert Borotkanics, Valery L. Feigin

**Affiliations:** ^1^School of Clinical Sciences, National Institute for Stroke and Applied Neurosciences, Auckland University of Technology, Auckland, New Zealand; ^2^Division of Arts, Law, Psychology & Social Sciences, School of Psychology, University of Waikato, Hamilton, New Zealand; ^3^Faculty of Science, School of Psychology, The University of Auckland, Auckland, New Zealand; ^4^UCL Research Department of Epidemiology and Public Health, University College London, London, United Kingdom; ^5^Faculty of Health and Environmental Sciences, Auckland University of Technology, Auckland, New Zealand

**Keywords:** mild traumatic brain injury, quality of life, behavior, emotional adjustment, social participation (MeSH), executive function, children

## Abstract

**Background:** Increasing evidence suggests potential lifetime effects following mild traumatic brain injury (TBI) in childhood. Few studies have examined medium-term outcomes among hospitalized and non-hospitalized samples. Study aims were to describe children's behavioral and emotional adjustment, executive function (EF), quality of life, and participation at 7-years following mild TBI using parents' and teachers' reports.

**Methods:** Nested case control study of 86 children (68% male, mean age at assessment = 11.27 years; range 7–17 years) who sustained a mild TBI 7-years previously, identified from a prospective, population-based study. They were compared to 69 children free from TBI (61% male, mean age at assessment = 11.12 years; range 5–17 years). In addition to parent-reported socio-demographic details, parents (mild TBI *n* = 86, non-TBI *n* = 69) completed age-appropriate standardized questionnaires about children's health-related quality of life, behavioral and emotional adjustment, EF, and social participation. Parents own mood was assessed using the Hospital Anxiety and Depression Scale. Teachers (mild TBI *n* = 53, non-TBI *n* = 42) completed questionnaires about children's behavioral and emotional adjustment, and EF.

**Results:** Parent reports showed median group-level scores for cases were statistically significantly greater than controls for emotional symptoms, conduct problems, hyperactivity/inattention, total behavioral difficulties, inhibitory control, shifting, planning/organizing, and Global Executive Composite (total) EF difficulties (*p*-values 0.001–0.029). Parent reports of child quality of life and social participation were similar, as were teacher reports of child behavioral and emotional adjustment, and EF (*p* > 0.05). When examining clinical cut-offs, compared to controls, cases had a higher risk of parent-reported total EF difficulties (odds ratio = 3.00) and, to a lesser extent, total behavior problems (odds ratio = 2.51).

**Conclusions:** As a group, children with a history of mild TBI may be at elevated risk for clinically significant everyday EF difficulties in the medium-term compared to non-TBI controls, as judged by their parents. Further multi-informant longitudinal research is required, following larger samples. Aspects requiring particular attention include pre-injury characteristics, such as sleep disturbances and comorbidities (e.g., headaches), that may act as potential confounders influencing the association between mild TBI and child behavioral problems.

## Introduction

Growing evidence from birth cohort studies suggests prospective links between a history of mild traumatic brain injury (TBI) in childhood and a range of risky behaviors later in life (1–3). These include increased risks for substance use, disruptive behavior disorders, conduct problems, and criminal behavior. A Swedish study of over 1 million adults found that having a mild TBI before the age of 25 years was associated with 1.18–1.52 risk ratios for low educational attainment, a psychiatric visit or hospitalization, receiving welfare, and/or drawing a disability pension (1). Similar associations between mild TBI and adverse outcomes are evident when injuries are sustained earlier during childhood and adolescence. Mild TBI between 6 and 15 years of age has been linked with increased arrests and property offenses at age 16–25 years (4). Growing evidence of associations between mild TBI in childhood and adverse long-term outcomes later in life raise questions about whether or not it is possible to detect indicators of maladjustment and difficulties in the medium-term following mild TBI. If so, it may be possible to provide additional support to help prevent, or lessen the likelihood of adverse long-term outcomes later in life. With more than doubled rates of TBI diagnosis over the past 10 years (5) and increasing healthcare use by patients with TBI (6), one approach to extending knowledge of medium-term outcomes is to examine children's well-being and development across multiple domains and settings.

Studies examining children's QoL following mild TBI offer mixed findings. Battista et al. study of children and adolescents with TBI included nine studies with four reporting good and five reporting poor QoL outcomes (7). Fineblit et al. systematic review of eight studies concluded that a small proportion of children had impaired health-related QoL (HRQoL) up to 1 year post injury or beyond (8).

Studies examining behavioral outcomes provide growing evidence of links between mild TBI in childhood and an increased presence of conduct problems (9), and attention deficit hyperactivity disorder (ADHD), a childhood onset neurobiological, neurodevelopmental disorder associated with increased risk for accidents and injuries (10). Interestingly, childhood mild TBI is commonly linked to increased rates of ADHD both prior to (11) and following mild TBI in terms of newly diagnosed cases (12). Yet, studies examining medium-term behavioral outcomes, including multi-informant reports and hospitalized and non-hospitalized TBI, are less common.

Another important domain to consider in light of evidence of at-risk behaviors later in life is children's social participation. The capacity to take part in everyday activities and to be included and accepted, is a key contributor to children's psychosocial growth (13), overall well-being (14), and positive development in adulthood (15). Yet, only a small number of studies have examined children's participation following mild TBI across multiple settings (i.e., home, school, and community). Two cross sectional studies of adolescent brain injury found that up to three-quarters of children and adolescents were restricted in their participation but both studies included children with a range of acquired brain injuries and did not include a non-TBI comparison group (16, 17). It is important to acknowledge that children's social participation may vary across different contexts (e.g., home vs. school). Where measurements are available, including reports from informants in different contexts, most often parents and teachers in child research (18), can provide greater insight into children's overall functioning.

One developmental domain that plays an important role in children's behavior, emotional control, and social participation is children's executive function (EF). Developing rapidly throughout childhood and adolescence (19), EF is a collective term for different cognitive processes (i.e., working memory, inhibitory control, planning) guiding cognitive, emotional, and behavioral functions (20). Healthy development of EF during childhood is a significant predictor for later life outcomes including physical health and personal finances (21). Two components of EF that may be especially related to risk-taking behavior are attention shifting [the ability to flexibly reallocate attention within one's internal and external environments to support goal-directed behaviors or meet task demands (22)] and inhibitory control [the ability to inhibit and override dominant responses and behaviors in favor of more appropriate responses (23)] (24). Evidence to date is mixed with some studies reporting EF deficits following mild TBI in childhood, especially in working memory (25, 26), while others do not (27, 28). Of note, most studies examining mild TBI samples have used decontextualized performance-based EF tests that may have limited ecological validity in terms of children's day-to-day EF (29). One exception is the parent report ecological study of EF using the Behavior Rating Inventory of Executive Function (BRIEF) by Sesma et al. (30). Results suggested that children hospitalized with mild, moderate, or severe TBI had significantly more EF difficulties compared to orthopedic controls at 3 and 12 months after injury. While noteworthy and consistent with links between TBI and risky behavior, these findings may not be generalisable to the broader population of children with mild TBI including non-hospitalized cases, which represent the majority of children who are diagnosed and treated outside of the hospital setting (31).

Using a nested case control design, study aims were to determine whether there were any statistically and/or clinically significant differences in parent report child HRQoL, behavior, everyday EF, and social participation (Aim 1), and teacher report child behavior and everyday EF (Aim 2) between children with a history of mild TBI (cases) and non-TBI controls.

## Materials and Methods

The study was conducted by inviting parents of all eligible children to complete a follow-up assessment either online or in-person. Most parents were seen in-person at a private residence. Parents were asked to provide contact details for each child's school teacher who was then invited to complete an online questionnaire. All parents provided informed written consent and assent was sought from child participants aged >7-years. The study was approved by the Northern Y Health and Disability Ethics Committee of New Zealand (NTY/09/09/095 and NTY/11/02/016), and the Auckland University of Technology Ethics Committee (AUTEC 09/265).

### Cases With Mild TBI

Cases were children (aged ≤ 17 years at follow-up) with mild TBI identified as part of the ‘Brain Injury Incidence and Outcomes In the New Zealand Community' (BIONIC) study, a population-based TBI incidence and outcomes study. Full details of the methodology of the BIONIC study, that took place in the Hamilton and Waikato Districts of New Zealand between 01st March 2010 to 28th February 2011, have been published separately (32). Mild TBI was defined as an acute brain injury resulting from mechanical energy to the head from external physical forces, with a Glasgow Coma Score of 13–15 and/or Post Traumatic Amnesia (<24 h) (33). Operationally, TBI was defined as including the presence of one or more of the following: (1) confusion or disorientation; (2) loss of consciousness; (3) post-traumatic amnesia; and (4) other neurological abnormalities (e.g., seizure) (34). Given the inherent difficulties in applying TBI criteria to children (i.e., determining confusion in young children), evidence of a head injury accompanied by medical/behavioral changes immediately following the injury were required to confirm TBI (e.g., vomiting, persistent crying). Case sample size was dictated by the number families of children with mild TBI identified in the BIONIC study who consented to take part in the 7-year follow-up.

### Non-TBI Controls

For comparison purposes, study controls were recruited from an existing control cohort to be similar to cases at a group level by sex and age, with current age similar by 6-month age bands. Controls had no previous history of TBI and were originally recruited when mild TBI cases were 12-months post-injury. Budget and logistical constraints meant that the controls were not recruited at the same time as cases. Controls were recruited between September 2011 to September 2013 by advertising at schools and businesses in the study area. On-going TBI-free status at the 7-year follow-up was determined by asking parents two questions: Had their child ever hit their head hard enough for them to seek medical attention? Had their child ever suffered a concussion or been knocked out? A study Diagnostic Adjudication Group reviewed instances where children's TBI history was unclear. We aimed for maximum recruitment of controls from an existing TBI-free cohort while intermittently monitoring group level matching to cases by sex and age.

### Measures

With the exception of TBI information relevant to cases only, follow-up assessment methods and measures were consistent across cases and TBI-free controls. Child (age, sex, area of residence, ethnicity), injury (TBI mechanism, prior TBI), and parent (age, sex, relationship to child, marital status, family SES, anxiety, and depression) details were based on parent-report and/or medical records. Potential confounders examined in the current study included family SES and parent mental health. Family SES (based on highest SES per family) at the time of follow-up was assessed using the Australian NZ Standard Classification of Occupations (ANZSCO), with classifications ranging from 1 = managerial, to 9 = unemployed. Parent self-report anxiety and depression were assessed using the 14-item Hospital Anxiety and Depression Scale (HADS) (35). Previously validated in mild TBI (36), higher scores (ranging from 0 to 21) indicate more anxiety or depression.

Parent and teacher report versions of the Strengths and Difficulties Questionnaire (SDQ) assessed children's hyperactivity/inattention, conduct problems, emotional symptoms, peer problems, prosocial behavior, and total behavior problems. Scoring was undertaken using SPSS syntax available via the SDQ website (www.sdqinfo.com). Higher scores indicate greater problems, except for the prosocial subscale where higher scores reflect better outcomes. The SDQ has discriminatory ability similar to other established measures of parent and teacher-reported child behavior (37, 38), and proven test-retest and internal reliability (39).

Age-appropriate, parent and teacher versions of the BRIEF (40) assessed children's inhibitory control, shift, emotional control, initiate, working memory, plan/organize, organization of materials, and monitor skills. The inhibit, shift, and emotional control subscales form a composite Behavioral Regulation Index. The other subscales form the composite Metacognition Index. A Global Executive Composite reflects scores from all subscales (herein referred to as total EF difficulties). Higher T-scores indicate more problems (mean score = 50, *SD* = 10). The BRIEF correlates significantly with the Conners Parent Rating Scale (41), has proven test-retest reliability, and good convergence/discriminance with the Child Behavior Checklist (42) and the Behavior Assessment System for Children (43).

A parent-report, age-appropriate version of the Pediatric Quality of Life (PedsQLTM) 4.0 Generic Core Scales (44–46) assessed child HRQoL. Each item, including reverse scoring, was rescaled on 0 to 100 scale (0 = 100, 1 = 75, 2 = 50, 3 = 25 and 4 = 0). Higher scores reflect better HRQoL. The PedsQL has established reliability and validity for use in pediatric populations, including TBI (47, 48).

The Child and Adolescent Scale of Participation (CASP) (49) assessed children's social participation at home, school, community compared to same-aged peers. Developed specifically for use with children aged ≥5 years following acquired brain injury, the CASP has proven construct validity and internal consistency within TBI populations (50). Higher scores indicate participation that is closer to that expected of their same aged peers.

### Analysis

Group characteristics were compared using descriptive statistics. For continuous variables we used *t*-tests and chi-square tests for categorical variables. Shapiro-Wilk tests supported the use of non-parametric tests as assumptions of normality were not met for parent and/or teacher report SDQ, BRIEF, PedsQL, and CASP scores (*p*s < 0.001). Descriptive statistics and Mann-Whitney U tests were performed to compare group median scores for parent and teacher reported outcomes. Group medians and interquartile ranges (IQR) showing the 25th and 75th percentiles are reported to indicate the distribution of data and to provide a reliable representation of central tendency (51). Score distributions of the controls were used to define clinically significant impairment (using a worst 10% cut-point). With prior use in child development studies (52, 53), including mild TBI (54), this approach increases measurement consistency and avoids problems associated with the use of test norms especially when measures are developed overseas. Chi-square analyses examined the proportions of cases and controls meeting clinically significant cut-offs for each composite score that differentiated the two study groups. Alpha level was 0.05 for all statistical tests. Cases and controls with missing data were excluded from related analyses. All analyses were completed using IBM SPSS for Windows version 26.0 (55).

## Results

### Study Sample

**Figure 1:**
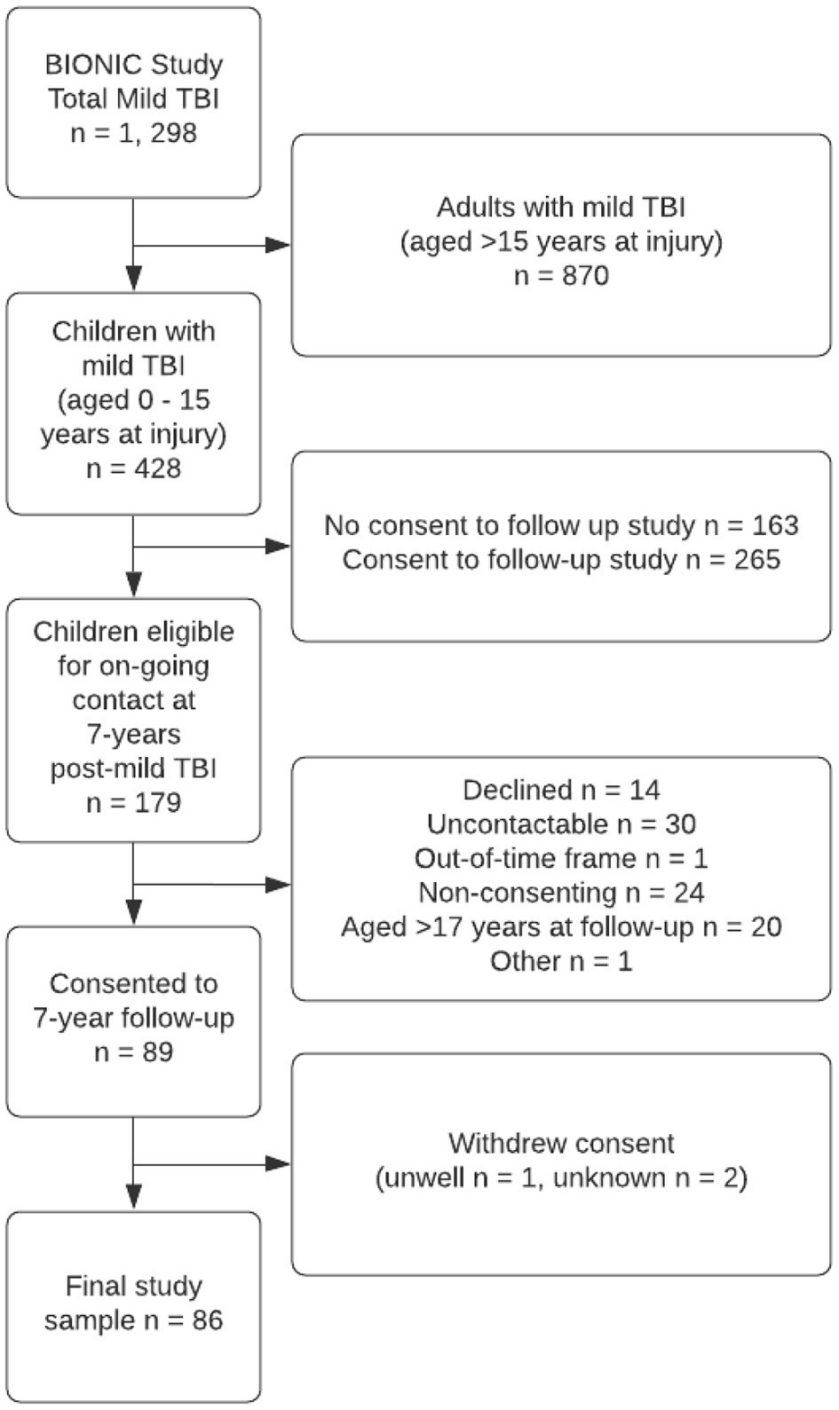
Case participation flow diagram.

As shown in Figure 1, cases included eighty-six children (aged 0–10 years at injury) who were followed-up at 7-years after mild TBI. As seen in Table 1, at follow-up, the mean age of children was 11.27 ± 2.81 years, the majority were male European and urban residents at the time of injury. Mild TBI cases were most commonly due to falls or exposure to mechanical force. Parents mean age at follow-up was 41.67 ± 6.75 years, and the majority were female, European, and married. Children with mild TBI who were included in the current analysis (*n* = 86) were compared to those from the BIONIC study cohort that had a mild TBI but who were not included (*n* = 342). Those included in the current analysis did not differ by sex (*p* = 0.31), but children included were more likely than those not included to be rural residents, of European ethnicity, and younger at the time of injury. Controls included sixty-nine children. Cases and controls did not differ in terms of child age, sex, ethnicity, and area of residence, nor parent age, sex, relationship to child, ethnicity, marital status, and mental health (Table 1). Cases were statistically significantly more likely to be of un/semi-skilled SES than TBI-free controls (*p* = 0.04).

**Table 1:** Sample characteristics for mild TBI cases and non-TBI controls.

	**Mild TBI cases (*n* = 86)**	**Non-TBI controls (*n* = 69)**	**Test of difference (mild TBI vs. TBI-free)**	***p***
**Child characteristics**				
Age (years)				
Mean (*SD*) age at injury	4.15 (2.82)	–	–	–
Mean (*SD*) age at follow-up [range]	11.27 (2.81) [7–17 years]	11.12 (2.97) [5–17 years]	*t*(*n* =153) = 0.32	0.74
Sex, *n* (%)				
Male	59 (68.6)	42 (60.9)	*t*(*n* =155) = 1.00	0.31
Female	27 (31.4)	27 (39.1)		
Ethnicity (7-years), *n* (%)				
Māori	15 (17.4)	9 (13.0)	χ^2^ (*n* = 155) = 0.57	0.74
Other	9 (10.5)	8 (11.6)		
NZ European	62 (72.1)	52 (75.4)		
Area of residence, *n* (%)				
Urban	57 (66.3)	56 (81.2)	χ^2^ (*n* = 155) = 4.29	0.03
Rural	29 (33.7)	13 (18.8)		
**Injury Factors**				
Mechanism of injury, *n* (%)				
Fall	56 (65.1)	–	–	–
Exposure to mechanical force	21 (24.4)	–	–	–
Traffic	4 (4.7)	–	–	–
Assault	4 (4.7)	–	–	–
Unknown	1 (1.2)	–	–	–
Prior TBI	15 (17.5)	–	–	–
**Parent characteristics (7-years)**				
Mean (*SD*) age (years)	41.67 (6.75)	42.17 (6.53)	*t*(*n* = 153) = −0.46	0.64
Sex (female), *n* (%)	81 (94.2)	62 (89.9)	χ^2^ (*n* = 155) = 1.00	0.31
Mother respondent, *n* (%)	79 (91.9)	63 (91.3)	χ^2^ (*n* = 155) = 3.37	0.18
European ethnicity, *n* (%)	78 (90.7)	62 (89.9)	χ^2^ (*n* = 155) = 0.03	0.86
Un/semi-skilled family SES^*^, *n* (%)	34 (41.5)	17 (25.8)	χ^2^ (*n* = 148) = 3.99	0.04
Married, *n* (%)	62 (72.1)	58 (84.1)	χ^2^ (*n* = 155) = 3.13	0.07
Mental health				
Mean (*SD*) anxiety^*†*^	5.12 (3.49)^*†*^	4.88 (4.02)	*t*(*n* =151) = 0.84	0.70
Mean (*SD*) depression^*†*^	2.81 (2.79)^*†*^	2.38 (3.06)	*t*(*n* =151) = 0.17	0.36

*n, sample size; SD, Standard deviation; TBI, traumatic brain injury; SES, socio-economic status at 7-years*.

* *Mild TBI n = 82 and TBI-free n = 66 due to missing 7 x data*.

† *Measured using the HADS and n = 84 due to 2 x missing data. Dash (–) indicates data not applicable*.

### Parent Report Child Outcomes (Aim 1)

As Table 2 shows, parent report revealed that cases had significantly higher median scores than controls across the following scales: emotional symptoms, conduct problems, hyperactivity/inattention, total behavior difficulties, inhibitory control problems, shift problems, plan/organize, behavioral regulation index, and total EF problems (*p* < 0.05). Cases and controls performed similarly on parent-reported measures of child HRQoL, peer problems, prosocial behavior, emotional control, working memory, organizing materials, and participation across home, community, and school settings (*p* > 0.05).

**Table 2:** Parent-reported child outcomes for mild TBI cases and non-TBI controls (Aim 1).

**Measure**	**Mild TBI cases** **(*n* = 86)** **Median (IQR)**	**Non-TBI controls** **(*n* = 69)** **Median (IQR)**	***Z*-value**	***P*-value**
**HRQoL (PedsQL Scales)**				
Physical	96.88 (90.63–100.00)	93.75 (87.50–100.00)	−1.607	0.108
Emotional	75.00 (60.00–90.00)	80.00 (60.00–90.00)	−0.221	0.825
Social	90.00 (75.00–100.00)	90.00 (80.00–100.00)	−0.125	0.901
School	77.50 (63.75–95.00)	85.00 (70.00–95.00)	−1.098	0.272
Psychosocial health summary	80.00 (69.58–90.42)	85.00 (71.67–91.67)	−0.742	0.458
Physical health summary	96.88 (90.63–100.00)	93.75 (87.50–100.00)	−1.607	0.108
Total HRQoL	84.78 (77.17–93.48)	86.96 (77.17–94.57)	−0.315	0.735
**Behavioral and emotional adjustment (SDQ Scales)**				
Emotional symptoms	2.00 (1.0–4.0)	1.00 (0.0–3.0)	−2.221	0.026
Conduct problems	1.00 (0.0–3.0)	0.00 (0.0–1.0)	−3.008	0.002
Hyperactivity/inattention	3.00 (1.0–6.0)	2.00 (1.0–3.5)	−2.702	0.007
Peer problems	1.00 (0.0–3.0)	1.00 (0.0–2.0)	−1.894	0.058
Prosocial behavior	9.00 (8.0–10.0)	9.00 (7.0–10.0)	−1.392	0.164
Total behavior difficulties	8.00 (4.0–14.25)	4.00 (2.5–8.0)	−3.244	0.001
**Executive function (BRIEF scales)**^***†***^				
Inhibit	48.00 (42.00–62.50)	44.00 (42.00–52.50)	−2.615	0.009
Shift	50.00 (41.50–61.50)	43.00 (38.00–52.50)	−3.059	0.002
Emotional control	49.00 (40.00–59.50)	46.00 (40.00–54.00)	−1.437	0.151
Initiate	52.00 (42.00–59.00)	47.00 (43.00–53.00)	−1.688	0.091
Working memory	52.00 (40.00–62.00)	46.00 (40.00–54.00)	−1.744	0.081
Plan/Organize	51.00 (43.00–61.00)	47.00 (41.00–53.00)	−2.286	0.022
Organization of materials	51.00 (43.00–60.50)	49.00 (43.00–55.00)	−0.764	0.445
Monitor	47.00 (37.00–57.50)	45.00 (37.50–50.50)	−1.549	0.121
Behavioral regulation index	48.00 (41.00–62.00)	44.00 (39.00–52.50)	−2.311	0.021
Metacognition index	49.00 (41.00–61.00)	46.00 (40.00–52.00)	−1.813	0.070
Global executive composite^*^	49.00 (41.00–61.00)	46.00 (40.00–50.00)	−2.190	0.029
**Social participation (CASP scales)**			
Home participation	100.00 (100.00–100.00)	100.00 (95.83–100.00)	−0.729	0.466
Community participation	100.00 (100.00–100.00)	100.00 (100.00–100.00)	−0.744	0.457
School participation	100.00 (100.00–100.00)	100.00 (100.00–100.00)	−1.401	0.161
Community living activities	100.00 (100.00–100.00)	100.00 (95.00–100.00)	−1.541	0.123

*n, study sample; IQR, Interquartile Range; HRQoL, Health-Related Quality of Life; SDQ, Strengths and Difficulties Questionnaire; BRIEF, Behavior Rating Inventory of Executive Function; CASP, Child and Adolescent Scale of Participation*.

† *Mild TBI n = 81, non-TBI controls n = 65 due to missing data*.

* *Mild TBI n = 79, non-TBI controls n = 64 due to missing data*.

### Teacher Report Child Outcomes (Aim 2)

As Table 3 shows, cases and controls performed similarly on all teacher report measures of child behavioral and emotional adjustment and EF in the school setting (*p* > 0.05).

**Table 3:** Teacher-reported child outcomes for mild TBI cases and non-TBI controls (Aim 2).

**Measure**	**Mild TBI cases** **(*n* = 53)** **Median (IQR)**	**Non-TBI controls** **(*n* = 42)** **Median (IQR)**	***P*-value**
**Behavioral and emotional adjustment (SDQ Scales)**			
Emotional symptoms	1.00 (0.00–2.00)	0.00 (0.00–1.00)	0.492
Conduct problems	0.00 (0.00–1.00)	0.00 (0.00–0.00)	0.064
Hyperactivity/inattention	3.00 (1.00–6.00)	2.00 (0.00–5.00)	0.051
Peer problems	1.00 (0.00–3.00)	0.00 (0.00–1.00)	0.076
Prosocial behavior	8.00 (5.00–9.00)	8.00 (6.75–10.00)	0.490
Total behavior difficulties	5.00 (2.50–11.50)	3.50 (1.00–6.25)	0.051
**Executive function (BRIEF Scales)**^***†***^			
Inhibit	46.00 (44.00–55.25)	45.00 (44.00–49.00)	0.397
Shift	47.00 (44.75–54.25)	47.00 (45.00–52.00)	0.845
Emotional control	46.50 (45.00–51.75)	46.00 (45.00–48.00)	0.702
Initiate	49.50 (43.00–63.00)	46.00 (43.00–55.00)	0.654
Working memory	48.00 (43.00–65.25)	46.00 (44.00–57.00)	0.438
Plan/Organize	48.00 (43.00–63.75)	47.00 (43.00–57.00)	0.916
Organization of materials	47.00 (44.00–57.00)	46.00 (44.00–51.00)	0.723
Monitor	49.00 (42.75–58.25)	48.00 (44.00–54.00)	0.584
Behavioral regulation index	48.00 (44.75–53.50)	46.00 (45.00–50.00)	0.392
Metacognition index	50.50 (42.75–64.00)	47.00 (44.00–55.00)	0.698
Global executive composite	49.00 (41.00–61.00)	46.00 (40.00–50.00)	0.385

*n, study sample; IQR, Interquartile Range; SDQ, Strengths and Difficulties Questionnaire; BRIEF, Behavior Rating Inventory of Executive Function*.

† *TBI n = 38, TBI-free n = 35 due to missing data*.

Given statistically significant group differences in family SES [a well-established mediator of children's development (56)] and lack of evidence of a confounding relationship with child outcomes, possible interaction effects between child outcomes, group status and family SES were further examined using generalized linear modeling (GLM) with a Gamma distribution and log link. Also known as a Gamma regression model, this approach provides robust estimates in the absence of normality. For this part of the extended analysis, rather than subscales scores, we used more robust total scores (SDQ total behavior difficulties score, and BRIEF Gobal Executive Composite score). Group status was coded as 0 = controls and 1 = cases. To broadly reflect family SES, this variable was recoded 1 = Professional/skilled (ANZCOS codes 1–3) and 0 = Semi/unskilled (ANZSCO codes 4–9). Despite group differences, area of residence was not entered into the model as urban or rural residence is not a well-established predictor of child psychosocial outcomes after mild TBI.

As Table 4 shows, group status (cases vs. controls) and family SES were significantly associated with parent report child total SDQ and BRIEF global executive composite scores. Children with mild TBI whose families had lower SES were more likely to be characterized by behavioral and EF difficulties than those children without mild TBI whose families had higher SES. There were no significant interactions between parent report of children's overall behavior difficulties or EF and group status and family SES. Therefore, having a history of mild TBI combined with being from a family of low SES did not appear to place children at heightened risk of poor behavioral and emotional adjustment and/or EF difficulties at 7-years post-injury.

**Table 4:** Gamma regression model results examining the roles of group status and family SES in child total outcome scores differentiating mild TBI cases and non-TBI controls.

			**95% CI**			
	**Coefficient (β)**	**(*SE*)**	***LL***	***UL***	**Wald chi-square**	***p***
**Parent report (Aim 1)**						
Total behavior difficulties (SDQ, *n* = 142)						
Intercept	2.739	(0.15)	2.443	3.036	328.569	<0.001
Group status (case, control)	−0.460	(0.12)	−0.702	−0.218	13.879	<0.001
Family SES (1-month)	−0.526	(0.15)	−0.0.83	−0.22	11.331	0.001
Total executive function difficulties (BRIEF GEC, *n* = 143)						
Intercept	59.333	(2.52)	54.390	64.277	553.319	<0.001
Group status (case, control)	−4.744	(1.82)	−8.319	−1.169	6.764	0.009
Family SES (1-month)	−8.345	(2.60)	−13.452	−3.238	10.257	0.001

*n, study sample; β represents the estimated average difference between the case and control groups; CI, Confidence interval; LL, lower limit; UL, upper limit; SDQ, Strengths and Difficulties Questionnaire; SES, Socio-economic status (reference group = professional/skilled); BRIEF GEC, Behavior Rating Inventory of Executive Function Global Executive Composite. Model coefficients are exponentiated to obtain rates of change per unit change of each independent variable*.

### Clinically Significant Child Outcomes (Aims 1 and 2)

As shown in Table 5, compared to non-TBI controls, parent report showed that mild TBI was associated with increased risk for clinically significant EF difficulties (* p* < 0.05) and, to a lesser extent, total behavior problems though between-group differences did not reach statistical significance (*p* > 0.05).

**Table 5:** The number (and percentage) of children meeting cut-offs for clinically significant^*^ problems in child total outcome scores differentiating mild TBI cases and non-TBI controls.

**Measure**	**Mild TBI cases (*n* = 86)**	**Non-TBI controls (*n* = 69)**	**OR (95% CI)**	***p***
**Parent report (Aim 1)**				
Total behavior difficulties (SDQ), *n* (%)	19 (22.1)	7 (10.1)	2.51 (0.988–6.384)	0.078
Total EF difficulties (BRIEF GEC^*†*^), *n* (%)	22 (27.8)	7 (10.9)	3.14 (1.245–7.937)	0.012

*n, study sample; SDQ, Strengths and Difficulties Questionnaire; BRIEF GEC, Behavior Rating Inventory of Executive Function Global Executive Composite; EF, Executive Function*.

* *Clinically significant defined as ≥ the 90th percentile of the non-TBI control group. OR denotes odds ratio. CI denotes confidence interval*.

† *Mild TBI n = 79, non-TBI controls n = 64 due to missing data*.

## Discussion

The present study aimed to determine whether there were any statistically and/or clinically significant differences in parent report child HRQoL, behavior, everyday EF, and social participation, and teacher report child behavior and everyday EF (Aim 2) between children with a history of mild TBI (cases) and non-TBI controls. The main finding of our nested case control study was that children with a history of mild TBI are more likely to be characterized by behavioral and emotional adjustment problems and EF difficulties in the home setting. While similar difficulties were not reported in the school setting, it is not uncommon for parents and teachers to differ in their impressions of children. Differences in reporting across home and school settings may arise due to differences in respondents' relationships with children and in their expectations. Parent reports may be influenced by prior knowledge of children's behavior before injury, while teachers may be comparing children to their classroom peers. Methodological reasons (i.e., statistical power) may also contribute to differences in parent and teacher reports, with fewer teacher respondents in the current study. Nevertheless, our findings highlight the added insight gained by examining multi-informant report which provides a more comprehensive portrayal of a child's current functioning (57). Parent report revealed that mild TBI was associated with more child emotional symptoms, conduct problems, hyperactivity/inattention, and overall behavior difficulties at home. Parent report also showed significantly more everyday EF difficulties among mild TBI cases compared to controls including but not limited to poorer inhibitory control and difficulties shifting attention. While teachers reported no statistically significant group differences in child behavior and everyday EF at school, the overall pattern of findings (i.e., direction of scores) suggests a trend toward greater difficulties among the mild TBI group. Parent report group differences were associated with group status and family SES. While there were no significant statistical interactions between child total outcome scores, group status and family SES, this pattern of findings suggests that children with mild TBI whose families had lower SES were more likely to be characterized by behavioral and EF difficulties than those children without mild TBI whose families had higher SES. However, having a history of mild TBI combined with being from a family of low SES did not appear to place children at heightened risk of poor behavioral and emotional adjustment and/or EF difficulties at 7-years post-injury.

These findings extend previous reports of associations between mild TBI in childhood and later adverse outcomes in hospitalized samples, particularly hyperactivity and inattention, (58) by identifying similar associations in a population-based sample. In the current study, parents reported statistically significantly greater behavioral and emotional adjustment difficulties among children with a history of mild TBI, particularly hyperactivity/inattention. Evidence of difficulties in the home environment suggests that additional support for children after mild TBI, even several years later, may be required. This might include interventions focused on improving attentional control, cognitive flexibility, planning and organizing, and goal setting. For example, children may benefit from help to generate alternative solutions to problems, establishing priorities and timeframes, and setting and managing realistic goals. While tested among mild complicated to severe TBI cases, pre-packaged, evidenced-based, multi-focal programmes specifically designed to support adolescent behavior and EF after TBI including Teen Online Problem Solving (TOPS) (59), and Counselor-Assisted Problem Solving (CAPS) (60) may be of assistance. Decontextualized interventions (i.e. drill-based skills training) may have limited generalizability to everyday contexts. However, symptom-specific interventions such as attention training [i.e., Attention Improvement and Management (AIM) program (61)] that integrate de-contextualized computerized drills with contextualized goal setting and strategies may also be helpful. Additional support to improve daily functioning may promote long-term improvements, especially in relation to EF skills that involve behavioral regulation (62) and have been linked to increased risk-taking behavior (23, 24).

Using an ecological assessment, the current study revealed poorer EF at 7-years post-injury across three of the four distinct domains of EF proposed by Anderson (19). Our findings revealed parent report difficulties with attentional control (inhibition), cognitive flexibility (shifting), and goal setting (planning). These findings are similar to those of Sesma and colleagues who, also using the parent report BRIEF, found more EF difficulties among children hospitalized with mild TBI compared to orthopedic controls at 3 and 12 months after injury (30). Together, these findings suggest that, as a group, hospitalized and non-hospitalized children with mild TBI may find it difficult to selectively attend to stimuli, regulate, and monitor actions so that plans are executed, shift between responses and learn from mistakes, and approach tasks in an efficient and strategic manner.

We also found that 22–30% of children with mild TBI met clinical criteria based on parent report of overall behavior problems and, moreso, everyday EF difficulties. These findings suggest that children with a history of mild TBI represent an at-risk group for difficulties in the medium-term post-injury. While cause and effect relationships cannot be inferred in the current study, a history of mild TBI may be a flag for possible behavioral and/or EF difficulties, regardless of whether or not these difficulties were present prior to or arose following mild TBI.

Based on parent report, children's behavioral and EF difficulties observed in the home setting do not appear to be adversely impacting their HRQoL and social participation at 7-years post-injury. However, it is possible that parents are more likely to report aspects of their child's functioning that have a more direct impact on the family (i.e., externalizing behaviors and EF) compared to HRQoL and social participation. Further, the measure of participation used in the current study tends to assess levels rather than the quality of children's social participation. It may be that parents continue to involve their child in activities at home, school, and in the community to the same extent as they did prior to mild TBI. However, the quality of children's social participation may be impacted by their behavior and EF. Future studies including child self-report may provide greater insight into associations between children's behavior, EF, HRQoL, and social participation several years after mild TBI.

Strengths of the current study are its inclusion of non-hospitalized cases that are often overlooked in previous studies, assessment of a broad range of outcomes, and use of multi-informant report—often considered the gold standard in assessing psychological outcomes. We also included a non-TBI control group. While preferable to examining mild TBI samples alone, we did not include an orthopedic injury control group that may be seen as a study limitation. Studies comparing children with mild TBI to healthy controls are more likely to find elevated rates of psychological and psychiatric problems than studies comparing to orthopedic controls. Comparing children with mild TBI to uninjured controls may fail to account for any generic impacts of injury (e.g., pain, medical treatment) (10). Relatedly, adverse outcomes are more prevalent in children with pre-existing difficulties (10). Our use of an uninjured comparison group may have overestimated group differences by failing to account for differences in preinjury status. However, it is worth noting that ADHD is associated with increased risk of injuries (not only TBI). Therefore, orthopedic controls might also be contaminated by a higher than usual rate of ADHD. It is also important to acknowledge the potential for recruitment and injury bias. Families (cases and controls) whose children had behavioral/emotional problems may be more likely to agree to participate. Further, while controls were systematically rescreened for TBI-free status at the 7-year time point, it is possible that undetected or undiagnosed mild TBI may have occurred that would impact the generalisability of study findings.

Compared to non-TBI controls, children with a history of mild TBI may represent an at-risk group for clinically significant everyday EF difficulties in the medium-term compared to non-TBI controls, as judged by their parents. Further multi-informant longitudinal research is required, following larger samples. Aspects requiring particular attention include pre-injury characteristics, such as sleep disturbances and comorbidities (e.g., headaches), that may act as potential confounders influencing the association between mild TBI and child behavioral problems.

## Data Availability Statement

The datasets presented in this article are not readily available due to ethical restrictions (consent was not sought from participants for data sharing). Requests to access the datasets should be directed to Kelly Jones, kelly.jones@aut.ac.nz.

## Ethics Statement

The studies involving human participants were reviewed and approved by Northern Y Health and Disability Ethics Committee of New Zealand (NTY/09/09/095 and NTY/11/02/016), and the Auckland University of Technology Ethics Committee (AUTEC 09/265). Written informed consent to participate in this study was provided by the participants' legal guardian/next of kin.

## Author Contributions

KJ, NS, SB-C, SA, AT, KP, RB, and VF contributed to conception and design of the study. KJ and AT organized the database. KJ and RB performed the statistical analysis. KJ wrote the first draft of the manuscript. RB wrote sections of the manuscript. All authors contributed to manuscript revision, read, and approved the submitted version.

## Conflict of Interest

The authors declare that the research was conducted in the absence of any commercial or financial relationships that could be construed as a potential conflict of interest.

## Publisher's Note

All claims expressed in this article are solely those of the authors and do not necessarily represent those of their affiliated organizations, or those of the publisher, the editors and the reviewers. Any product that may be evaluated in this article, or claim that may be made by its manufacturer, is not guaranteed or endorsed by the publisher.
